# Shifting Paradigms in TNBC Treatment: Emerging Alternatives to Capecitabine in the Post-Neoadjuvant Setting

**DOI:** 10.3390/curroncol31070278

**Published:** 2024-06-30

**Authors:** Halima Abahssain, Amine Souadka, Rania Alem, Matteo Santoni, Nicola Battelli, Eric Amela, Antoine Lemaire, Joseph Rodriguez, Hassan Errihani

**Affiliations:** 1 Oncology and Medical Specialties Department, Valenciennes General Hospital, 59300 Valenciennes, France; raniaalem19@gmail.com (R.A.); amela-y@ch-valenciennes.fr (E.A.); 2 Equipe de Recherche en Oncologie Translationnelle (EROT), University Mohammed V in Rabat, Rabat 8007, Morocco; a.souadka@um5r.ac.ma (A.S.); errihani5@gmail.com (H.E.); 3 Surgical Oncology Department, National Institute of Oncology, University Mohammed V in Rabat, Rabat 8007, Morocco; 4 Centre d’Investigation Clinique, Centre Hospitalier Universitaire Ibn Sina, Rabat 6527, Morocco; 5 Oncology Department, National Institute of Oncology, University Mohammed V in Rabat, Rabat 8007, Morocco; 6 Oncology Unit, Macerata Hospital, 62100 Macerata, Italy; matteo.santoni82@gmail.com (M.S.); nicola.battelli@sanita.marche.it (N.B.); 7 Supportive Care Department, Valenciennes General Hospital, 59300 Valenciennes, France; lemaire-a@ch-valenciennes.fr (A.L.); rodriguez-j@ch-valenciennes.fr (J.R.)

**Keywords:** triple-negative breast cancer, capecitabine, immunotherapy, PARP inhibitors, antibody–drug conjugates, neoadjuvant therapy, adjuvant therapy

## Abstract

Background: Triple-negative breast cancer (TNBC) remains a clinically challenging subtype due to its aggressive nature and limited treatment options post-neoadjuvant failure. Historically, capecitabine has been the cornerstone of adjuvant therapy for TNBC patients not achieving a pathological complete response (pCR). However, the integration of new modalities such as immunotherapy and PARP inhibitors has prompted a re-evaluation of traditional post-neoadjuvant approaches. Methods: This review synthesizes data from pivotal clinical trials and meta-analyses to evaluate the efficacy of emerging therapies in the post-neoadjuvant setting. We focus on the role of immune checkpoint inhibitors (ICIs), PARP inhibitors (PARPis), and antibody-drug conjugates (ADCs) alongside or in place of capecitabine in TNBC treatment paradigms. Results: The addition of ICIs like pembrolizumab to neoadjuvant regimens has shown increased pCR rates and improved event-free survival, posing new questions about optimal post-neoadjuvant therapies. Similarly, PARPis have demonstrated efficacy in BRCA-mutated TNBC populations, with significant improvements in disease-free survival (DFS) and overall survival (OS). Emerging studies on ADCs further complicate the adjuvant landscape, offering potentially efficacious alternatives to capecitabine, especially in patients with residual disease after neoadjuvant therapy. Discussion: The challenge remains to integrate these new treatments into clinical practice effectively, considering factors such as drug resistance, patient-specific characteristics, and socio-economic barriers. This review discusses the implications of these therapies and suggests a future direction focused on personalized medicine approaches in TNBC. Conclusions: As the treatment landscape for TNBC evolves, the role of capecitabine is being critically examined. While it remains a viable option for certain patient groups, the introduction of ICIs, PARPis, and ADCs offers promising alternatives that could redefine adjuvant therapy standards. Ongoing and future trials will be pivotal in determining the optimal therapeutic strategies for TNBC patients with residual disease post-neoadjuvant therapy.

## 1. Introduction

Neoadjuvant chemotherapy was initially considered as a strategy for breast-conserving surgery in locally advanced and inflammatory breast cancer and has significantly evolved [1]. This strategy showed that patients who had a pathological response had better survival. Studies correlating neoadjuvant chemotherapy response with survival across early breast cancer subtypes, especially in aggressive types like triple-negative breast cancer (TNBC) and Her2-positive cancers, have highlighted the importance of achieving a pathological complete response (pCR) [2]. An international analysis involving over 11,900 patients demonstrated that pCR attainment leads to significantly improved survival, particularly in these aggressive subtypes [3]. Consequently, neoadjuvant approaches have become the standard of care in Her2-positive cancers and TNBC for cT1c and/or node positive [4].

Several trials have reinforced neoadjuvant strategies, incorporating treatments beyond standard anthracyclines and taxanes to enhance pCR and thereby improve survival outcomes. 

Until recently, standard neoadjuvant chemotherapy was based on anthracyclines and taxanes. A benefit on DFS and OS was shown with a dose-dense regimen [5].

The newly validated neoadjuvant strategies include carboplatin and immunotherapy to anthracycline and taxane neoadjuvant therapy [6].

Poggio et al. confirmed that carboplatin-based neoadjuvant chemotherapy increased the pCR rate significantly from 37.0% to 52.1%; however, no survival benefit was demonstrated. As a result, adding platinum to neoadjuvant chemotherapy has been considered an option in the neoadjuvant setting for TNBC [7].

Immune checkpoint inhibitors (ICIs) have shown their role in the TNBC neoadjuvant strategy. Indeed pembrolizumab is validated as a standard of care in stage II and III TNBC in the neoadjuvant setting [6].

However, post-neoadjuvant therapy escalation has been proposed to improve survival for patients who do not achieve pCR. In TNBC subgroups lacking hormone receptors and Her2 overexpression, chemotherapy remains the primary option for those not achieving pCR. Capecitabine, an oral prodrug of fluorouracil, has been a cornerstone in post-neoadjuvant care for non-pCR TNBC patients [8].

The CREATE-X trial [8], a pivotal phase III study, evaluated disease-free survival (DFS) as a primary endpoint with capecitabine in Her2-negative residual invasive breast cancer. The trial showed significant improvements in DFS and overall survival (OS) for patients treated with capecitabine. 

However, recent advancements in immunotherapy and PARP inhibitors necessitate a re-evaluation of capecitabine’s role. Despite its established efficacy, the lack of direct comparisons with newer therapies and potential biases due to differences in patient populations and treatment regimens highlight the need for further research.

These new options did not integrate capecitabine in their strategy because corresponding studies commenced before capecitabine’s validation in the adjuvant setting for non-pCR early TNBC, leaving uncertainties about capecitabine’s role in this context with the new treatments.

This review aims to expose the management complexity of residual disease after neoadjuvant therapy in TNBC, review the role of capecitabine inside the dominant impact of validated immunotherapy and PARP inhibitors, and investigate the promising role of ADCs.

## 2. Capecitabine in TNBC Residual Disease after NACT

For many decades, observation was the adopted attitude in post-neoadjuvant therapy, after surgery and/or radiotherapy, knowing that patients who did not achieve pathological complete response had a 20 to 30% relapse risk [9]. Dominant strategies focused on reinforcing neoadjuvant therapies to maximize pCR. Indeed, three studies, GeparSixto, CALGB 40603, and BrighTNess, showed that the addition of carboplatin to standard neoadjuvant chemotherapy increased the rate of pCR but, at the same time, enhanced hematological toxicities [10,11,12].

Capecitabine is an oral prodrug of fluorouracil and has been validated for several years as a second-line chemotherapy in metastatic breast cancer [13].

Capecitabine was the only validated drug in the adjuvant setting for patients with residual disease. The CREATE-X study, a phase III trial, evaluated disease-free survival (DFS) as a primary endpoint with capecitabine in Her2-negative residual invasive breast cancer following neoadjuvant chemotherapy with anthracyclines, taxanes, or both, compared to the control arm. A total of 32.2% of patients were classified as having TNBC, and approximately 40% had clinical stage IIIA or IIIB. The patients received six to eight cycles of capecitabine at a dose of 1250 mg/m^2^ of body surface area, twice per day, on days 1 to 14 every 3 weeks [8].

The final analysis showed an improvement in DFS compared to the control arm, with 74.1% vs. 67.6% of patients alive and free from recurrence or second cancer at 5 years (HR = 0.70, 95% CI = 0.53–0.92, *p* = 0.01). Overall survival, a secondary endpoint, was also improved in the capecitabine group compared to the control group (89.2% vs. 83.6% alive at 5 years; HR 0.59; 95% CI, 0.39 to 0.90; *p* = 0.01) [8]. Since its approval in 2017, capecitabine has been considered standard adjuvant care for residual disease after neoadjuvant chemotherapy in early TNBC.

Other hypotheses have been tested, particularly the potential sensitivity of some types of TNBC to platinum therapy [14]. The EA1131 trial attempted to demonstrate the superiority of four cycles of platinum therapy (carboplatin or cisplatin) every 3 weeks in post-neoadjuvant treatment of TNBC with residual disease over six cycles of capecitabine 1000 mg/m^2^ b.i.d. on days 1–14. The study was initially designed to compare platinum therapy to placebo; however, after the availability of capecitabine, the EA1131 trial was amended to replace placebo with capecitabine.

The study failed to demonstrate the benefit of platinum therapy over capecitabine in patients with residual disease after neoadjuvant therapy. Moreover, the experimental arm was associated with more toxicities [15].

Thoughtful issues have been raised about worse disease-free survival in capecitabine-treated patients in the EA1131 trial than in the CREATE-X trial and about patients who might benefit from adjuvant platinum therapy. Differences in populations and treatments may contribute to outcome differences. Indeed, the EA1131 trial selected more high-risk patients with Residual Cancer Burden [RCB] (II–III) than the CREATE-X trial, 100% and 58%, respectively. 

In addition, the CREATE-X trial population was exclusively Asian; however, 20% of the EA1131 trial participants were black; these participants’ breast cancer prognoses and drug metabolisms were different.

Capecitabine dose and tolerance were also different, in fact, in the CREATE-X trial, capecitabine was received at 1250 mg/m^2^ twice daily, and 80% of patients completed eight cycles. However, in the EA1131 trial, capecitabine was used at 1000 mg/m^2^ twice daily for six cycles and about 20% of patients needed dose delays or adjustments.

## 3. Immunotherapy and PARP Inhibitors Era

The treatment landscape for TNBC has undergone significant changes with the introduction of immunotherapy and PARP inhibitors. These novel therapies have been integrated into neoadjuvant settings and hold potential in the adjuvant stage for certain patient subgroups.

Immunotherapy, specifically immune checkpoint inhibitors (ICIs), has shown promising results. The KEYNOTE-522 trial is a pivotal study demonstrating the efficacy of pembrolizumab, an ICI, in combination with chemotherapy. It showed a notable increase in pCR rates and event-free survival (EFS) when added to standard neoadjuvant chemotherapy regimens for stage II and III TNBC [6].

Similarly, PARP inhibitors have emerged as a valuable option, particularly for patients with BRCA mutations. These inhibitors are considered in the adjuvant setting of post-neoadjuvant therapy for non-pCR TNBC patients with BRCA mutations, offering a targeted approach to treatment [16].

These advancements necessitate a re-evaluation of capecitabine’s role. Initially a cornerstone for post-neoadjuvant care in non-pCR TNBC, capecitabine now faces competition from these newer modalities. The key question is whether capecitabine remains the standard of care in the adjuvant setting, or if its role is diminished or altered in the face of these emerging therapies.

**Figure 1:**
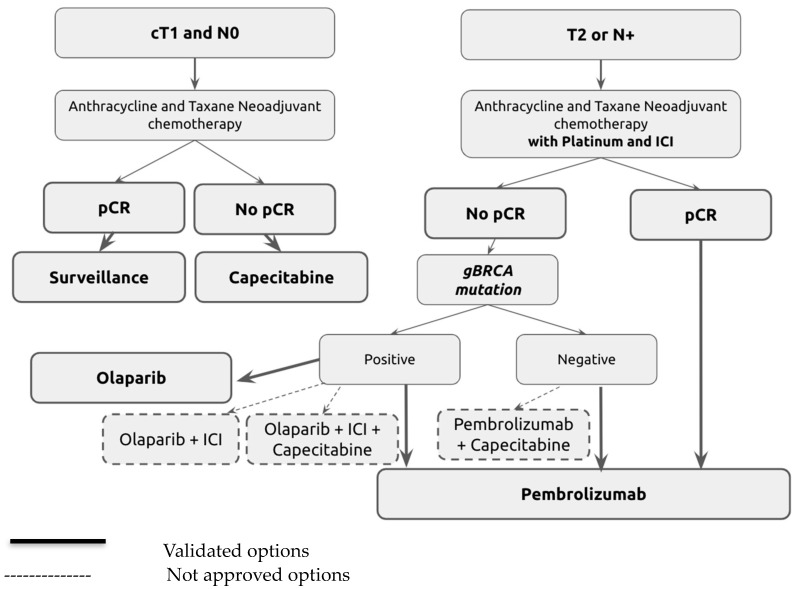
Adjuvant treatment algorithm for triple-negative breast cancer (TNBC) following neoadjuvant therapy.

In daily practice, adjuvant therapy for patients with residual disease after neoadjuvant therapy based on the KN522 protocol in stage II and III TNBC represents an oncological challenge. Decisions mainly depend on BRCA mutation status (Figure 1).

Ongoing trials aim to investigate the opportunities of the combination of validated therapies to add survival benefits based on the safety and efficacy of these combinations in other locations.

### 3.1. Immunotherapy in TNBC Residual Disease

Patients with stage II and III TNBC receive neoadjuvant chemo-immunotherapy according to KN522 [6]. Patients randomized in the pembrolizumab arm received systematic adjuvant pembrolizumab regardless of response to neoadjuvant therapy (complete response or residual disease). The study shows that the magnitude of benefit is numerically greater in patients with residual disease who were in the pembrolizumab arm than those in the placebo arm (3-year EFS 67.4% vs. 56.8%, HR 0.70, 95% CI 0.52–0.95) [6]. However, patients with residual disease had a substantial residual risk than patients with complete response, and no treatment escalation had been proposed for these patients to compensate for survival benefit. This study was started before capecitabine’s validation in non-pCR TNBC.

The main questions in this situation concern the impact of adding capecitabine in non-pCR non-mutated BRCA TNBC after neoadjuvant treatment according to the KN522 plan. Patients in the CREATE-X trial did not receive either carboplatin or pembrolizumab in the neoadjuvant setting, so it is uncertain whether capecitabine can add benefit to patients receiving the KN522 protocol. However, in the absence of other new options in this population, capecitabine can be considered [17]. Another question that remains unanswered until validation in clinical trials concerns the safety of the association of capecitabine and pembrolizumab. This combination was tested in head and neck and gastro-esophageal cancer and is considered a safe standard of care [18,19,20].

Many studies are ongoing to evaluate the impact of capecitabine in association with immunotherapy. SWOG S1418/NRG-BR006 is an ongoing phase III randomized study comparing pembrolizumab to observation after surgery in patients with residual cancer after neoadjuvant chemotherapy, particularly triple-negative cancers and more than 1 cm of residual invasive cancer or positive lymph nodes (>pN1mic) [21]. Capecitabine was used in the adjuvant setting with pembrolizumab initiation after capecitabine completion [21]. The OXEL trial (NCT03487666) and MIRINAE trial (NCT03756298) are ongoing phase II studies comparing Nivo vs. capécitabine vs. Nivo + capecitabine and Atez + capecitabine vs. capecitabine, respectively, after NACT including an anthracycline or a taxane, or both [22,23].

BreastImmune03 (NCT03818685), a phase II study, evaluates the clinical benefit of post-operative treatment associating radiotherapy + nivolumab + ipilimumab vs. radiotherapy + capecitabine for TNBC patients with residual disease (Table 1).

### 3.2. PARP Inhibitors in TNBC Residual Disease

BRCA are tumor suppressor genes that encode proteins implicated in homologous recombination repair. Germline BRCA mutations can be found in 10–15% of TNBC and are responsible for alterations in repair mechanisms [24]. PARP inhibitors represent a targeted therapy able to block double-strand break (DSB) repairs through the homologous recombination pathway in patients with mutated BRCA through synthetic lethal effects [25]. This therapy was initially tested in the metastatic breast cancer (MBC) setting and confirmed by OlympiAD and EMBRACA trials [26,27]. In the adjuvant setting, the OLYMPIA trial has validated PARP inhibitors in adjuvant high-risk Her2-negative breast cancer with germline BRCA mutation [16].

Patients with TNBC were included if tumor size was more than 2 cm or if lymph node involvement after adjuvant chemotherapy or no pCR was detected after neoadjuvant chemotherapy including taxane, anthracyclines, or both. This study shows that the addition of olaparib in non-pCR TNBC improves DFS (85.9% vs. 77.1%, HR 0.68; 95% CI 0.50 to 0.91; *p* = 0.0091) and OS (HR 0.68; 98.5% CI 0.47 to 0.97; *p* = 0.009). Due to the presence of a strong antigenic driver, it seems evident that olaparib should be introduced in the adjuvant setting in BRCA-mutated non-pCR TNBC.

However, the neoadjuvant strategy in KN522 in patients with stage II and III TNBC includes pembrolizumab and carboplatin to taxane and anthracycline, which is different from neoadjuvant therapy in the OLYMPIA trial containing only taxane and anthracycline. Another discussion point concerns the lack of information about germline BRCA mutations in KN522; however, what appears relevant and attractive is to test if the combination of olaparib and pembrolizumab is safe and can improve survival in patients with residual disease and germline BRCA mutation after KN522 neoadjuvant therapy.

Immunotherapy and PARP inhibitors have shown synergistic activities in preclinical studies [28]. PARP inhibitors block single-strand DNA repair, leading to DNA damage, increased tumor mutational burden, and the potential to enhance the response to immunotherapy [28]. 

In locally advanced breast cancer with tumor BRCA mutation, the TOPACIO (KEYNOTE-16) and MEDIOLA trials are single-arm phase II studies that found that the association of ICIs with PARP inhibitors enhances the overall response rate [29,30].

In early breast cancer TNBC subgroup analysis, the phase I-II SPY trial also showed improvement in overall response rate among patients with neoadjuvant association of ICIs, PARP inhibitors, and paclitaxel compared to paclitaxel [31]. The association of PARP inhibitors and ICIs has proven its safety and the majority of toxicities are related to PARP inhibitor therapy [29]. These studies did not explore ICI and PARPi association with PARP inhibitors alone. It appears evident that adjuvant PARP inhibitors in non-pCR mutated BRCA TNBC after the KN522 plan are an optimal option in light of the strong survival benefit and the presence of a target driver. However, the benefit of the association of PARPis and pembrolizumab has to be confirmed.

Many questions remain unanswered in patients with residual disease and germline BRCA mutation concerning the benefit of the association of PARP inhibitors and ICIs, the comparison between PARP inhibitors and capecitabine, the combination of capecitabine and olaparib, as well as the association of ICIs, olaparib, and capecitabine. The eventual survival benefit, toxicities management, and the cost difference between the use of all the possible combinations of these therapies have to be considered. Further randomized studies are needed to confirm these theories.

## 4. Future Opportunities with ADCs

Antibody–drug conjugates (ADCs) have recently transformed the treatment landscape of many cancers, especially breast cancer. They are composed of a three-part drug: a monoclonal antibody (mAb), a linker, and a cytotoxic payload. 

After ADC molecule administration, the antibody component of the ADC recognizes and binds to cell-surface antigens highly expressed in target cancer cells. The ADC–antigen complex is internalized and the cytotoxic payload is released in a bioactive form inside the cell leading to cell death.

The primary goal is to deliver drugs inside cancer cells with a high concentration using a targeted cell receptor.

Compared to conventional chemotherapy, this mechanism of action is more effective, less toxic, and has better pharmacokinetics, pharmacodynamics, and biodistribution.

Other mechanisms of action include reinforcing the immune system through antibody-mediated cellular toxicity, tumor-specific immunity, and adaptive immune responses [32].

In metastatic breast cancer (MBC), trastuzumab emtansine, trastuzumab deruxtecan, and sacituzumab govitecan are cornerstone therapies. Trastuzumab emtansine is a standard adjuvant therapy for Her2-positive residual disease after neoadjuvant therapy [33].

Sacituzumab govitecan (SG) is an ADC with SN-38, an active metabolite of irinotecan, coupled with a humanized IgG antibody that targets Trop-2, a transmembrane glycoprotein highly expressed in TNBC. SG is approved for patients with metastatic TNBC who have received ≥2 prior systemic therapies based on progression-free survival (PFS) and overall survival (OS) benefits demonstrated in the ASCENT study [34].

Ongoing studies are comparing ADCs with capecitabine or the combination of capecitabine and immunotherapy to evaluate their impact in the adjuvant setting in patients with residual disease after neoadjuvant chemotherapy (NACT) (Table 2).

SG is under evaluation in the SASCIA trial, a phase III, prospective, international, multi-center, randomized study in patients with Her2-negative breast cancer (BC) and residual disease after NACT. Patients are randomized to receive either sacituzumab govitecan or treatment of the physician’s choice (capecitabine or platinum-based chemotherapy). Patients who received immunotherapy in the neoadjuvant setting are allowed to be included.

ASCENT-05 (NCT05633654) is another phase III study evaluating sacituzumab govitecan and pembrolizumab vs. the treatment of the physician’s choice (pembrolizumab or pembrolizumab + capecitabine) in patients with triple-negative breast cancer (TNBC) who have residual invasive disease after surgery and neoadjuvant therapy. TROPION-Breast03 (NCT05629585) is a phase III study testing another ADC, Dato-DXd, which also targets Trop-2, with or without durvalumab vs. the investigator’s choice of therapy (capecitabine, pembrolizumab, or pembrolizumab + capecitabine) in patients with stage I-III TNBC without pathological complete response after neoadjuvant therapy (Table 2). 

Trastuzumab deruxtecan (T-DXd) is an ADC-targeting Her2 that has changed the landscape of Her2-positive metastatic breast cancer. In addition to its activity in Her2 overexpression, T-DXd has shown activity in tumors with lower levels of Her2 expression, without amplification, overexpression, or both, now referred to as ‘Her2-low’. Indeed, T-DXd has improved progression-free and overall survival compared to the physician’s choice of chemotherapy in DESTINY-Breast04 and is considered a validated option in Her2-low MBC [35].

The Her2-low subgroup represents more than 50% of tumors initially considered Her2-negative, including TNBC [36].

Given the major benefit in metastatic situations, T-DXd seems to be a promising ADC in the neoadjuvant or residual disease in the post-NACT setting in TNBC and Her2-low early breast cancer. This potential benefit needs to be elucidated in clinical trials. 

These ADCs have reached phase III clinical trial in the adjuvant setting in residual disease TNBC prior to eventual approval in clinical practice. In addition, important ongoing axes of investigations concern ADC engineering to select the best target antigens, linkers, and payloads. Indeed, areas of innovation focus on producing performant molecules with high DAR (drug-to-antibody ratio); radionuclide-conjugated ADCs to deliver selective radioactive drugs; developing ADCs with molecules stimulating the immune system or with two different payloads to the same mAb; conjugation of payloads to bispecific Abs to target different pathways; and selecting the best antigens to be targeted to spare normal cells. 

Potential biomarkers such as ctDNA clearance in non-pCR TNBC after NACT can be a future strategy to select patients who will benefit from these innovative therapies.

## 5. Socio-Economic Challenges with New Therapies

New therapies like PARP inhibitors (PARPis), immune checkpoint inhibitors (ICIs), and antibody–drug conjugates (ADCs) represent significant advancements in TNBC treatment. However, their high costs pose challenges, particularly in low- and middle-income countries (LMICs) where access to genetic testing, consultations, and advanced cancer medications is often limited [37].

The World Health Organization’s Model Lists of Essential Medicines lag in including these newer cancer therapies, exacerbating disparities in global health equity [38].

Health insurance and coverage models significantly impact the accessibility of these expensive therapies. In countries with universal health coverage, essential cancer medications may be provided at little to no cost. Conversely, in systems with minimal or absent health coverage, patients often face prohibitive out-of-pocket expenses, leading to treatment abandonment and poorer outcomes [39].

Regulatory hurdles and complex approval processes also limit access. The registration and approval of cancer medicines in many LMICs are restricted by complex, inefficient, and costly processes [39,40].

This not only delays access to new treatments but also contributes to the overall cost burden.

Ensuring equitable access to safe, effective, affordable innovative cancer therapies represents a highly challenging situation. Indeed patients, healthcare systems, and the government have to guarantee reasonable medicine prices to fix health issues and, in return, pharmaceutical industries have to secure their optimal benefits to pursue investment in research on the development of new and efficient therapies to improve patients’ survival. 

Many policy approaches with the potential to improve access to expensive but innovative therapies have been adopted by both wealthier countries and LMICs. For example, tiered pricing adjusts the entry price of a supplier’s product based on the income or ability to pay in the country where the medicine is sold. In this case, prices are proportional to the target market income, higher in higher-income countries, and lower in lower-income countries [41].

Another policy approach involves managed-entry agreements (MEAs), also known as risk-sharing agreements, between pharmaceutical companies and payers. These agreements provide coverage for innovative therapies while controlling risk and managing uncertainty over their effectiveness or budgetary impact. The goal is to balance the trade-off between delayed access at a lower cost vs. early access at a higher cost and high risk [41].

Additionally, proposing strategies for optimizing resource allocation in resource-constrained countries can help overcome barriers to accessing innovative therapies. 

A patient-centric approach can also help adapt treatment to individual patient profiles. By selecting treatments based on genetic, clinical, and socio-economic factors, outcomes can be optimized and toxicities minimized, ensuring a more equitable and effective treatment strategy.

### Recommendations

Advocating for international collaborations and policy reforms is crucial. These efforts should focus on reducing the cost of cancer medications, streamlining regulatory processes, and enhancing global access to these treatments. Indeed, innovative financing models, such as risk-sharing agreements between pharmaceutical companies and payers, and strategies for optimizing resource allocation in resource-constrained settings, can be released by involving international organizations that can provide subsidies to LMICs in order to reduce innovative treatment costs. LMICs can also lower costs by acting on import duties, giving additional funding to the healthcare sector, and negotiating with laboratories through purchasing centers. A research collaboration to reduce the treatment cost by developing a bioequivalence treatment is another solution.

## 6. Conclusions

As we navigate the evolving terrain of TNBC treatment, the role of capecitabine, a long-standing cornerstone in post-neoadjuvant care, is being re-evaluated in the face of emerging therapies like PARPis and ICIs. The ongoing clinical trials and research into novel treatment modalities, including ADCs, are poised to further reshape the TNBC treatment landscape. 

While these new treatments offer promising results, their integration into standard care protocols is not without challenges, particularly in socio-economic and regulatory contexts.

Consequently, there is a pressing need for global and deep thinking to increase accessibility and address the disparities by developing an efficient financial model. This includes fostering international collaborations, policy reforms, and innovative funding models to enhance the availability and affordability of advanced therapies.

From a clinician’s perspective, adapting to these changes involves not only staying abreast of the latest research but also advocating for policies and practices that put patient welfare at the forefront, particularly in resource-limited settings.

The diagram in Figure 1 illustrates the treatment pathways based on patient response and genetic status post-neoadjuvant therapy. Initial treatment includes anthracycline and taxane, with additional platinum and immune checkpoint inhibitors (ICIs) for higher-stage or node-positive disease. Post-treatment routes are guided by the pathological response (pCR or non-pCR) and genetic markers such as gBRCA mutations. Solid lines indicate validated treatment options, while dotted lines represent strategies not yet universally approved, highlighting areas of ongoing research and potential clinical application.

### Chemotherapy Regimens

- Anthracycline and Taxane Neoadjuvant Chemotherapy: four cycles of dose-dense AC60 or EC90 (doxorubicin 60 mg/m^2^ or epirubicin 90 mg/m^2^, plus cyclophosphamide 600 mg/m^2^) every 14 days followed by 12 weekly paclitaxel 80 mg/m^2^.
- Anthracycline and Taxane Neoadjuvant Chemotherapy with Platinum and ICIs (KN522 Protocol): Weekly paclitaxel (80 mg/m^2^) and carboplatin (AUC five every 3 weeks, or 1.5 once weekly) for the first 12 weeks, plus four cycles of pembrolizumab (200 mg) followed by four cycles of doxorubicin (60 mg/m^2^) or epirubicin (90 mg/m^2^), plus cyclophosphamide (600 mg/m^2^) administered once every 3 weeks and four cycles of pembrolizumab (200 mg).
- Adjuvant Capecitabine: Oral capecitabine at a dose of 1250 mg/m^2^, twice per day, on days 1 to 14 every 3 weeks for six or eight cycles.
- Adjuvant Olaparib: Oral olaparib 300 mg twice per day for 1 year.
- Adjuvant Pembrolizumab: Pembrolizumab at a dose of 200 mg for nine cycles.

## Figures and Tables

**Table 1:** Trials involving capecitabine with ICIs in Her2-negative BC with residual disease after NACT.

Study Title	Trial Phase	Population	Treatment Arms	Primary Outcome
SWOG S1418/NRG-BR006	Phase III	TNBC with residual disease after NACT. Residual disease is defined as ≥1 cm residual invasive carcinoma in the breast or positive lymph nodes (ypN1mi-3).	Adjuvant pembrolizumab for 1 year Observation Capecitabine was used in the adjuvant setting with pembrolizumab initiation after capecitabine completion.	Invasive DFS
OXEL trial (NCT03487666)	Phase II	TNBC with residual disease after NACT. Residual disease is defined as ≥1 cm residual invasive carcinoma in the breast or macroscopically positive lymph nodes.	Nivo Capecitabine Nivo + capecitabine	Changes in a peripheral immunoscore at week 6
MIRINAE trial (NCT03756298)	Phase II	TNBC with residual disease after NACT. Residual disease is defined as ≥1 cm residual invasive carcinoma in the breast or macroscopically positive lymph nodes.	Atez + capecitabine Capecitabine	Invasive DFS
BreastImmune03, NCT03818685	Phase II	TNBC with residual disease after NACT. Residual disease is defined as an RCB score of II or III.	Nivolumab + Ipilimumab + radiotherapy Capecitabine + radiotherapy	DFS

**Table 2:** Trials including capecitabine with ADCs in Her2-negative BC with residual disease after NACT.

Study Title	Trial Phase	Population	Treatment Arms	Primary Outcome
SASCIA trial (NCT04595565)	Phase III	Her2-negative BC with residual disease after NACT. * Residual disease is defined as follows: (1) macroscopic residual disease in the primary tumor (ypT > 1 mm) for TNBC; (2) CPS + EG ≥ 3 or CPS + EG 2 and ypN+ for HR + BC.	Sacituzumab govitecan. Treatment of physician’s choice (capecitabine or platinum-based chemotherapy).	Invasive DFS
ASCENT-05, NCT05633654	Phase III	Residual invasive disease after surgery and neoadjuvant therapy.	Sacituzumab govitecan + pembrolizumab. Treatment of physician’s choice (pembrolizumab or pembrolizumab + capecitabine).	Invasive DFS
TROPION-Breast03 NCT05629585	Phase III	Stage I-III triple-negative breast cancer without pathological complete response following neoadjuvant therapy.	Dato-DXd, with or without Durvalumab. Investigator’s choice of therapy (capecitabine, pembrolizumab, or pembrolizumab + capecitabine)	iDFS for Dato-DXd + durvalumab vs. ICT

## Data Availability

The data presented in this study is available in this article

## Abbreviations

TNBC	Triple-negative breast cancer
NACT	Neoadjuvant chemotherapy
pCR	Pathological complete response
DFS	Disease-free survival
MBC	Metastatic breast cancer
PARPi	Poly (ADP-ribose) polymerase inhibitor
ICI	Immune checkpoint inhibitor
Dato-DXd	Datopotamab deruxtecan
AUC	Area under the curve
SG	Sacituzumab govitecan
T-DXd	Trastuzumab deruxtecan
